# A Comparative Study on the Pain Threshold Experienced by Fibromyalgia Patients Following Acute SARS-CoV-2 Infection

**DOI:** 10.3390/life14080942

**Published:** 2024-07-27

**Authors:** Daniela Matei, Rodica Traistaru, Anca Maria Amzolini, Laura Simona Ianosi, Carmen Daniela Neagoe, Adina Mitrea, Diana Clenciu, Taina Elena Avramescu

**Affiliations:** 1 Department of Medical Rehabilitation, University of Medicine and Pharmacy Craiova, 200349 Craiova, Romania; mateidana30@yahoo.com (D.M.); rodicatraistru@gmail.com (R.T.); 2 Department Medical Semiology, University of Medicine and Pharmacy Craiova, 200349 Craiova, Romania; 3 Department of Dermatology, University of Medicine and Pharmacy Craiova, 200349 Craiova, Romania; simona.ianosi@umfcv.ro; 4 Department of Internal Medicine, University of Medicine and Pharmacy Craiova, 200349 Craiova, Romania; daniela.neagoe@umfcv.ro; 5 Department of Diabetes, Nutrition and Metabolic Diseases, University of Medicine and Pharmacy Craiova, 200349 Craiova, Romania; diana.clenciu@umfcv.ro; 6 Sport Medicine and Physiotherapy, University of Craiova, 200585 Craiova, Romania; taina_mistico@yahoo.com

**Keywords:** fibromyalgia, SARS-CoV-2 infection, pain threshold, non-pharmacological treatment, algometry

## Abstract

Significant gaps remain in the understanding of the etiology and pathogenesis of fibromyalgia (FM), and the COVID-19 pandemic has introduced even more unknowns. Social factors specific to that period, the viral infection itself, and/or vaccination are additional elements that can complicate the progression of the disease or the response to treatment. Aim: The primary hypothesis to be evaluated in this study is that an acute COVID-19 infection, even when considered recovered, may induce changes in the response to non-pharmacological treatment in FM patients, particularly concerning pain. Results: We included 128 patients diagnosed with FM before the pandemic began. The patients were divided based on their history of acute SARS-CoV-2 infection and COVID-19 vaccination status. All patients followed the same rehabilitation program (cognitive therapy, kinesitherapy). Perceived pain: The non-COVID-19 patient groups showed a statistically significant reduction in pain at the final evaluation compared to patients with a history of acute SARS-CoV-2 infection (*p* < 0.001). Algometric evaluation: Patients without COVID-19 infection and that were vaccinated exhibited the best improvement in pain threshold, both across evaluation times (*p* < 0.001) and compared to any of the other three groups studied (*p* < 0.001). Using the WHYMPI questionnaire, the same group of patients (those not having experienced acute COVID-19 and who were vaccinated) was the only group with a statistically significant improvement in pain severity (*p* = 0.009). In conclusion, to control and improve FM pain symptoms, in addition to appropriate medication, we propose paying additional attention to the history of acute SARS-CoV-2 infection and the COVID-19 vaccination status.

## 1. Introduction

In 1972, Smythe first described fibromyalgia (FM) as a disease marked by soft tissue pain, stiffness, and localized painful sensitivity, despite normal examination results. Hench introduced the term “fibromyalgia” in 1976, emphasizing muscular pain [1]. The identification of FM was based on the triad of generalized chronic pain, painful tender points, and non-restorative sleep, leading to the development of the initial diagnostic criteria [2,3,4]. These criteria evolved over the years until the American College of Rheumatology (ACR) established a new set in 1990 [5], which was later updated in 2010 to address its limitations [6].

Studies in the United States suggest a primary care prevalence of 3.4%, while a European Community study estimated an overall prevalence of 4.7% [7]. The women to men ratio for FM varies from 7:1 [7] to 10:1 [8]. Regarding the age at which the disease most frequently appears, the peak onset is typically between 30 and 50 years old, with the fifth decade of life being the most affected [9].

While significant gaps persist in understanding the causes and development of FM [10], it is proposed that a combination of biological, psychological, and social factors contributes to the amplification of pain and increased sensitivity of the central nervous system to peripheral stimuli [11].

This mechanism is indicated by the deficient inhibitory control of the brain over non-painful repetitive somatosensory stimuli [12] and its exaggerated, atypically intense response to painful stimuli [13]. Central sensitization is a form of neural hyperexcitability that results in increased perception of pain in the area subjected to nociceptive stimulation (hyperalgesia), pain in areas near and distant to the stimulated region (referred pain), and pain in unstimulated areas (allodynia) [14]. Triggers for FM may include viral or bacterial infections, acute illnesses, physical traumas, surgeries, motor vehicle accidents, or stressful psychosocial circumstances [15].

The onset of the novel coronavirus disease 2019 (COVID-19) led governments worldwide to implement lockdown measures to curb the spread of the virus. However, these widespread lockdowns significantly impacted the psychological and emotional well-being of the general population, resulting in a notable worsening of symptoms among FM patients, including increased pain, anxiety, sleep disruptions, and depression [16,17]. Studies by Aloush et al. (2021) and Batres Marroquín et al. (2022) reported a significant worsening of FM symptoms during the COVID-19 pandemic [18,19]. It is widely recognized that over three-quarters of individuals with fibromyalgia experience chronic fatigue, a symptom that exacerbates disability in FM [4,20]. Additionally, a significant number of FM patients also qualify for a diagnosis of chronic fatigue syndrome (CFS) [21]. Similarly, the long-term effects of (severe acute respiratory syndrome) SARS, such as fatigue, mirror those seen in fibromyalgia patients. While post-COVID-19 fatigue syndrome may stem from damage to olfactory sensory neurons [22], the root cause in FM seems to be linked to alterations in motor unit recruitment strategies [23].

Factors such as social distancing, economic issues, and difficulties in accessing medical services contributed to a decline in the perception of the disease. Some researchers suggest that the SARS-CoV-2 virus may directly affect the central and peripheral nervous systems, leading to musculoskeletal and psychological disturbances [17]. However, studies investigating the potential role of COVID-19 as a trigger for FM are lacking. Given that COVID-19 is an infectious disease of viral origin, and its associated syndrome is notably linked with myalgia, it is reasonable to hypothesize a possible connection between COVID-19 and the worsening of FM [24]. Conversely, the process of vaccination, acting both as an inflammatory trigger and a psychologically stressful event, may present a particular challenge for individuals with FM [25].

Due to the complex array of symptoms experienced by individuals with FM, no single therapeutic approach has proven fully effective. Regarding the pharmacological treatment for fibromyalgia, most of the drugs approved by the American Food and Drug Administration (FDA), such as duloxetine, milnacipran, and pregabalin, only provide partial relief of the symptoms. No medication is currently capable of controlling all the symptoms completely [26]. Among non-pharmacological treatment options, medical rehabilitation methods show significant promise and yield positive results in managing FM symptoms [27,28]. Currently, according to EULAR guidelines, adapted physical therapy is the primary treatment option, backed by strong evidence [29]. The objectives of physical activity and exercise training are to enhance physical fitness and function, alleviate symptoms, and improve overall health and well-being [30]. Moldofsky was the first researcher to observe a link between pain and exercise, showing that deep sleep deprivation lowered the pain threshold in sedentary individuals, but not in those who were more physically fit. He demonstrated that fit individuals were less likely to develop FM symptoms when their deep sleep stages (3 and 4) were intentionally disrupted [31]. The first exercise intervention for FM was published in 1988, and numerous clinical trials have been conducted since then [32].

Cognitive-behavioral therapy (CBT), developed in the mid to late 20th century, is the most effective psychological intervention for treating FM and its associated symptoms. Modern CBT is grounded in the cognitive theory of emotional responses, which posits that dysfunctional thinking leads to pathological negative emotions, influenced by the patient’s belief system. CBT can reduce pain and fatigue, improve emotional well-being, elevate mood, and potentially enhance physical and social functioning [33]. The benefits of this therapy are attributed to the reduction of hyperalgesia and pain-related catastrophizing, as evidenced in FM patients. The CB model asserts that affective, behavioral, cognitive, and sensory or physical aspects are all crucial for understanding a patient’s experience of pain, emphasizing the significant role of individual beliefs on the pain experience [34].

Given the uncertainty regarding the pathogenesis of FM and its wide range of symptoms, treating this disease remains challenging. The main hypothesis to be evaluated is that an acute SARS-CoV-2 infection, even after recovery, may alter the response to non-pharmacological treatment in FM patients, particularly in terms of pain. The primary objective is to determine the extent to which a recovered COVID-19 infection influences pain perception in patients undergoing a standard regimen of cognitive therapy combined with physical therapy. The secondary objective is to assess whether COVID-19 vaccination status should be considered as an additional factor in evaluating the therapeutic response.

## 2. Materials and Methods

### 2.1. Design Overview

An interventional prospective cohort study was conducted in the Department of Medical Rehabilitation and Internal Medicine Clinics, in hospitals affiliated with the University of Medicine and Pharmacy of Craiova, Romania, between May 2022 and January 2024. We included FM patients attending outpatient clinics who had been diagnosed with FM before the beginning of the pandemic. Participant eligibility was established, and baseline data were collected. The patients were stratified by prior acute SARS-CoV-2 infection status and vaccination status, with eligible subjects being divided into four groups.

The trial consists of three visits over 28 weeks:

- The initial interview (*Pre*) contained general data: age, marital status, occupation, area of residence, behavioral data, history of the disease, associated disorders. In the same interview, the patients gave a description of the pain regarding localization, intensity, frequency, and evolution from the beginning of the disease, previous treatments, and psychological factors. In addition to the initial interview and evaluation protocol, patients underwent three tests: a subjective pain assessment using a visual analogue scale, an objective pain level measurement using algometry, and the West Haven-Yale Multidimensional Pain Inventory (WHYMPI);
- Following this, patients attended 12 sessions of individualized non-pharmacologic therapy. At the end of the treatment (*Post*), subjects were evaluated in the same manner as at the time of admission;
- The patients were also scheduled for a follow-up (*F-up*) evaluation, 24 weeks after the end of the treatment.

### 2.2. Study Arms

Eligible patients met the following criteria: (1) adults over 18 years of age, (2) signed an informed consent form to participate, (3) had a confirmed previous diagnosis of FM according to the 2010 ACR criteria, (4) spoke and understood the language perfectly, (5) had no prior exposure to kinesiotherapy (aerobic exercises) and cognitive behavioral therapy, and (6) were on a stable dose of analgesic treatment for at least one month prior to study inclusion and maintained constant analgesic treatment throughout the entire treatment period. None of the patients included in the study received drugs such as duloxetine, milnacipran, and pregabalin, as they are not approved for the treatment of FM in Romania.

The exclusion criteria were: (1) uncooperative patients, (2) illiteracy, (3) alcoholism, (4) severe psychiatric disorders, (5) associated conditions that might otherwise explain the evolution of the symptoms, or (6) diagnosis of long COVID/post-COVID-19 syndrome or post-acute sequelae of COVID-19.

In all the patients, we assessed the history of SARS-CoV-2 infection, confirmed by a positive polymerase chain reaction test on respiratory samples, or a positive antigen test during the acute phase of the disease according to medical records, or by the presence of antibodies against SARS-CoV-2 in unvaccinated patients, or a vaccination status with two doses of an mRNA vaccine supported by medical documentation.

Based on the predetermined inclusion and exclusion criteria, eligible patients were informed about the study and provided appropriate informed consent. After obtaining informed consent from the patients, we divided them into 4 different groups:

- FM patients non-COVID-19, non-vaccinated (*FM non-COV*, *non-Vacc*) (*n* = 32), comprising unvaccinated FM patients without a history of COVID-19 infection;
- FM patients COVID-19, vaccinated with 2 doses of an mRNA vaccine (*FM COV*, *Vacc*) (*n* = 32), including vaccinated FM patients who had recovered from SARS-CoV-2 infection at any time since the beginning of the pandemic, but no later than 6 months prior;
- FM patients COVID-19, non-vaccinated for SARS-CoV-2 (*FM COV*, *non-Vacc*) (*n* = 32), consisting of non-vaccinated patients with a history of past COVID-19 infection, not more recent than 6 months;
- FM patients non-COVID-19, vaccinated for SARS-CoV-2 (*FM non-COV*, *Vacc*) (*n* = 32).

Patients who attended at least 70% of the program sessions were included in the statistical analysis. Thus, the final number of patients in the first group (*FM non-COV*, *non-Vacc*) was 30, in the second group (*FM COV*, *Vacc*) was 30, in the third group (*FM COV*, *non-Vacc*) 29 of the initially included patients, and in the fourth group (*FM non-COV*, *Vacc*) 31 patients met the aforementioned criterion.

### 2.3. Treatments/Interventions

All patients participated in programs that included non-pharmacological treatment, CBT, and a kinesiotherapy (KT) regimen for 4 weeks, and they were instructed by the same team members. Regardless of the group they belonged to, the patients attended 12 sessions for both CBT and KT (three sessions per week). Each session lasted 2 h for CBT and 1 h for KT.

*The KT intervention* consisted of two types of sessions: postural hygiene and aerobic exercise. The training program lasted 4 weeks and took place in the rehabilitation gym, in groups of up to 12 patients. Each session was conducted and supervised by both a rehabilitation physician and at least one KT specialist. Initially, each session lasted about 20 min, gradually increasing in duration and intensity over the weeks to a maximum of one hour. Each session began with a warm-up and included exercises for the central axis of the body, as well as the upper and lower limbs, concluding with breathing and relaxation exercises. Most exercises were presented with two alternatives, depending on each patient’s physical condition.

The aerobic exercise sessions aimed to establish the goals of the program, increase awareness of how physical activity influences each patient’s symptoms, perform exercises while monitoring the difficulties and ease of execution for each patient, and improve their balance and physical condition.

The postural hygiene sessions aimed to identify incorrect postures that can lead to increase pain over time, teach proper postures and offer advice for protecting the back and major joints during activities of daily living, promote symmetric and stable postures, provide correct proprioceptive information and an accurate body scheme and image.

*The CBT* included techniques for relaxation and pain management. It also involved restructuring occupational performance and gradually increasing activity. Additionally, it focused on identifying problems that arise in performing life roles and explaining aspects that influence complaints in FM. Each intervention group consisted of 10 to 12 participants. Each session employed cognitive-behavioral techniques through methods such as relaxation and breathing exercises, visualization, and addressing misconceptions.

### 2.4. Ethical Considerations

This study was performed in accordance with the principles of the Helsinki Declaration and Good Clinical Practice and was approved by the Ethics Committee of the University of Medicine and Pharmacy of Craiova (no. 81/13.05.2022). All the patients provided written informed consent.

### 2.5. Assessments/Measurements

Pain assessment was conducted using the following methods:

- *Intensity Rating*: Pain intensity was rated on a numeric scale from 0 (no pain) to 10 (maximum intensity) [35]. Patients were asked to rate the highest, lowest, and average levels of pain;
- *Algometry*: It was performed using a digital algometer from Somedic SenseLab. The device applies pressure to tissue at a constant rate until the patient reaches their pain threshold. The algometer includes a round probe sensor connected to a pressure transducer and a patient-operated switch. It offers three probe sensor sizes and five slope options (pressure application rates). The patients pressed the switch when they started to feel pain, allowing the device to record a numeric value (in kilopascals). The evaluator applied pressure with the algometer until the patient pressed the switch, at which point the value was displayed on the LCD screen and recorded. The algometer was set to B3 (using a 1 square centimeter probe and a 30 kilopascals/second slope). It was applied at a right angle to the evaluation area and stabilized between the investigator’s second and third fingers. Measurements were taken at three points on each side of the body (left and right): C5 vertebra, the second metacarpal space, 6 cm below the tibial tuberosity on the side of the calf in the anterior tibial muscle. The patient lay prone to allow for measurements at the cervical points and in a supine position, with relaxed arms and extended legs for other points. Three measurements were taken at each of the six points with a 10 s break between them, and the order of evaluation was established for each patient. The device was calibrated, as per the manufacturer’s instructions, before use. This method followed the recommendations of the International Association for the Study of Pain;
- *West Haven-Yale Multidimensional Pain Inventory (WHYMPI):* This inventory consists of three parts [36]:
  1. *Perceived Pain Intensity and Impact*: Evaluates pain intensity and its impact on various life aspects (work, social life, mood, family, household activities) through 20 items divided into five sections, namely interference, support, pain severity, life control, and affective distress;
  2. *Responses of Significant Others*: Assesses the patient’s perception of how significant others (e.g., spouse) respond to their pain communication (ignoring, helping, distracting) through 14 items combined into three sections, namely negative responses, solicitous responses, and distracting responses;
  3. *Activity Frequency*: Measures the frequency of the patient’s performance of common activities (washing dishes, going out, grocery shopping, social and recreational activities, driving, cooking) through 18 items resulting in five sections, namely household chores, outdoor work, activities away from home, social activities, and general activity.

Each item was scored on a Likert scale from 0 (not at all) to 6 (very much). In the present study, the results from Part I of the questionnaire were included, as this section is most relevant to the other two methods of pain assessment. The result was the arithmetic mean of the answers to the questions in that section.

### 2.6. Statistical Analysis

The Statistical Package for Social Sciences (SPSS) version 20 (IBM Corporation, Chicago, IL, USA) was employed to create and analyze the database. Given that the sample sizes exceeded 30 subjects, the assumptions of normality and homoscedasticity were first checked. The Kolmogorov–Smirnov test, which tests for normality with a null hypothesis of normal distribution, was used. This hypothesis is rejected if the significance level is 0.05 or less. Most variables met the normality assumption, and for those that did not, the standard error of kurtosis was considered. Despite some variables not meeting the normality criteria, none had extreme values warranting exclusion from parametric tests. Homoscedasticity was assessed using the Levene test, which tests for the homogeneity of variances. The null hypothesis is rejected if the significance level is 0.05 or less. Descriptive analyses and comparisons of the means and percentages were performed to characterize the samples, both interventional and control samples. The descriptive analysis provided context for the samples within relevant sociodemographic and clinical parameters. ANOVA was used to analyze potential differences in continuous and nominal variables between the two samples. Differential analysis for each group assessed the evolution of variables over time. For each group, paired sample *T*-tests and repeated measures were used to compare the means at three evaluation points (*Pre*, *Post*, and *F-up*). Variance analysis was conducted using independent sample *T*-tests and ANOVA, with the Huynh–Feldt correction applied when sphericity was not assumed. The interpretation of statistical significance (*p*-value) for the differences in variables between the treatment options, evaluation moments, or correlation tendencies was based on the significance threshold.

## 3. Results

### Demographic Characteristics

During the study period, 128 FM patients were initially included. However, eight patients did not attend at least 70% of the program sessions and were excluded from the statistical analysis. Table 1 shows that the final sample consisted of 120 participants, aged between 25 and 56 years. The majority were females (>93%), with secondary education and medically retired. There were no statistically significant differences between the four groups in terms of age or sex distribution (*p* > 0.05). Regarding patient education, no statistically significant differences were observed, except for higher education. For this category, there was a slight statistical significance between the studied groups (*p* = 0.03), with a highly significant difference between Groups I and II (*p* = 0.003). Most patients in the study were either unemployed or medically retired, with statistically significant differences (0.01 < *p* < 0.05) observed only for the medically retired status between Groups II and III compared to either Group I or IV.

The review of medical histories revealed that patients reported experiencing pain for over six years before being diagnosed with FM, with an average disease duration of seven years before study inclusion. The most common comorbid condition was anxiety or depression, affecting approximately 75% of the participants, followed by sleep disturbances and tension headaches, symptoms found in more than half of the patients included in the study. There were no statistically significant differences in these comorbidities among the studied groups. Table 2 details the differences in comorbidities between the groups.

**Figure 1:**
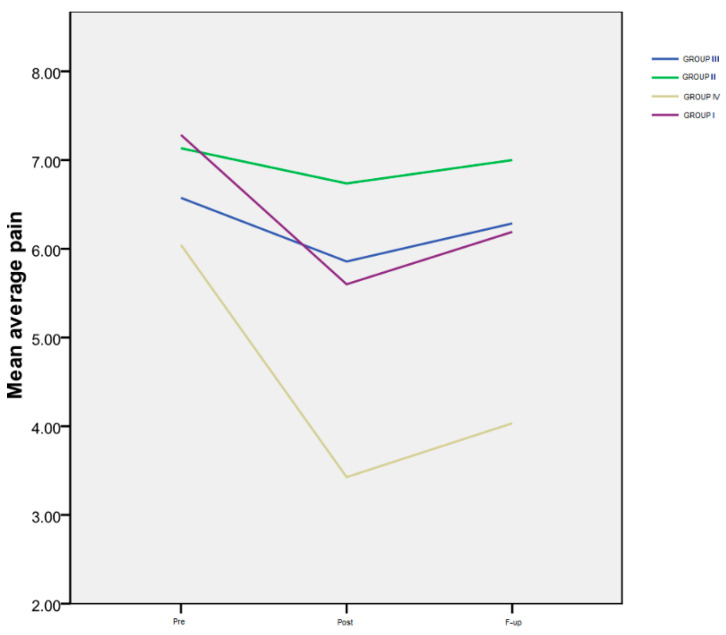
Average pain.

The results of the *average pain level* rated by the patients on a numeric scale (VAS), for all four monitored groups, are shown in Table 3 and Figure 1.

*Time progression:* Group I exhibited a notably significant difference between the *Pre* and *Post* assessments (*p* < 0.001), as well as between the *Pre* evaluation and the *F-up* (*p* < 0.001), and between the *Post* and the *F-up* assessment (*p* < 0.001). For Group IV, a marked decrease was observed between the *Pre* and *Post* evaluations (*p* < 0.000), as well as between the *Pre* evaluation and the *F-up* (*p* < 0.001). However, there was no significant difference between the *Post* evaluation and the *F-up* (*p* = 0.1). Notably, there was no statistically significant difference in the temporal progression of this variable for the second and third groups.

*Inter-group differences*: Regarding the interactions between groups (Figure 1), there were no significant differences among the four groups at the *Pre* evaluation, indicating that all the patients had a similar level of pain at the beginning of the study. However, at the *Post* assessment, a highly significant difference was observed between Group IV and Group II (*p* < 0.001), as well as between Group IV and Group III (*p* < 0.001). Additionally, there was a significant difference between Group I and the patients in Group II (*p* = 0.014), but not between Group I and Group III (*p* = 0.58). At the *F-up* evaluation, the average pain level reported by the patients in Group IV was significantly lower than that of the patients in Group I (*p* = 0.03), and even more reduced compared to both Group III (*p* < 0.001) and Group II (*p* < 0.001).

**Figure 2:**
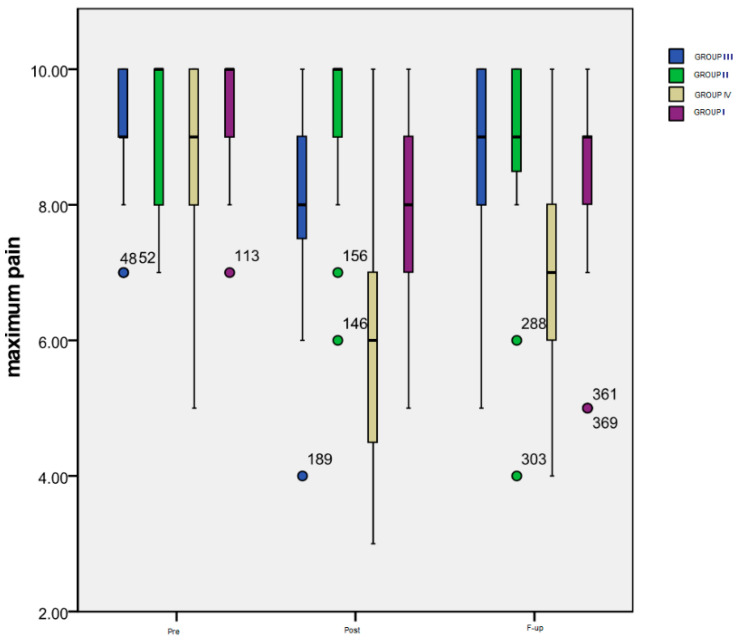
Evolution of maximum pain level.

Figure 2 summarizes the evolution over time of the *maximum pain level* rated by the patients of all four groups on a numeric scale.

*Time progression:* As it can be seen in the chart, the most important variation was that of the patients included in Group IV: a significant decrease (*p* < 0.001) between the maximum pain level at the *Pre* (mean = 8.56) and *Post* (mean = 5.82) evaluation, followed by a significant increase (*p* = 0.002) from *Post* to *F-up* (mean = 6.93) moments, but maintaining an important difference (*p* < 0.001) between *Pre* and *F-up*. The only significant difference (*p* = 0.003) for the patients included in Group III is the one between the *Pre* (mean = 9.13) and *Post* (mean = 7.94) evaluation, while for the subjects from Group II there was no significant difference registered. Group I also experienced a very significant decrease (*p* < 0.001) between *Pre* (mean = 9.53) and *Post* (mean = 8.08) moments, and between *Pre* and *F-up* (mean = 8.5) evaluations (*p* < 0.001), the increase between *Post* and *F-up* also being statistically significant (*p* = 0.019).

As for the *inter-group differences*, there were significant ones noted at *Post* monitoring between Group IV and III (*p* = 0.002), Group IV and Group II (*p* < 0.001), Group I and Group II (*p* = 0.013) and, finally, between the subjects from Group I or Group IV (*p* < 0.001). At the *F-up*, significant differences were maintained between Group IV and Group III (*p* = 0.007), between Group IV and Group I (*p* = 0.001), and between Group IV and Group II (*p* = 0.007).

The means and standard deviations of the measured *pain threshold* for the four groups are presented in Table 4. The table includes the values obtained from all measured areas of the body (on the right-R, as well as on the left side-L), detailed according to each evaluation moment.

*Time progression:* The most significant differences were noted for Group IV between the *Pre* and *Post* evaluation for all the monitored areas: the right cervical area (*p* = 0.001), the left cervical area (*p* = 0.014), the right hand (*p* = 0.036), the left hand (*p* = 0.009), the right tibial area (*p* < 0.001), and the left tibial area (*p* = 0.001). Between the *Pre* and *F-up* evaluations, significant differences could still be seen for the following areas: right cervical (*p* = 0.003), left hand (*p* = 0.01) and, especially, for both right and left tibial areas (*p* < 0.001).

*Inter-group differences*: There was a highly significant difference (*p* < 0.001) observed at all assessment points between Group IV patients and every other group, both at the end of the treatment application (*Post*) and at 6 months after the cessation of the applied non-pharmacological therapy (*F-up*), as is shown in Table 5. No statistically significant differences were observed among the groups of patients who had COVID-19, regardless of their vaccination status, at any of the subsequent evaluation moments. Referring to Group I, compared to either Group II or III, statistical significance was only evident when considering two out of the three evaluated regions (cervical and hands), and the difference lost its significance at the *F-up* when compared to Group II (*p* > 0.05).

**Figure 3:**
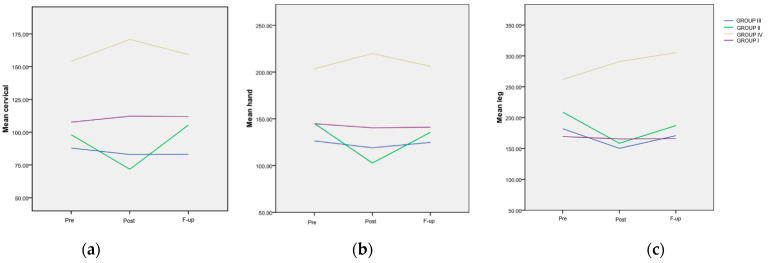
The evolution of the mean painful threshold, differentiated, in various areas and treatment groups. (**a**) Evolution of the mean values in the cervical area; (**b**) evolution of the mean values in the hand area; (**c**) evolution of the mean values in the tibial area, in regard to the maximum pain level.

The trend of the *mean values from the cervical area* for the four groups is depicted in Figure 3a. A highly significant difference (*p* < 0.001) was observed at all assessment points between Group IV and every other group. The evolution of the *mean values from the hand area* for the four groups is also illustrated in Figure 3b. Similar to the findings in the cervical area, a highly significant difference (*p* < 0.001) was observed at all assessment points between patients included in Group IV and any other group. The trend of the *mean values from the tibial area* for the four groups is presented in Figure 3c. A significant difference was noted at the *Post* evaluation between Group IV and every other FM group (*p* < 0.001). Additionally, a significant difference was recorded at the *F-up* evaluation between the mentioned group and every other FM group.

Strong correlations were found between *the three areas subjected to algometry,* as follows:

- Cervical and hand areas: r = 0.826, *p* < 0.001 (r = 0.768 *Pre*; r = 0.865 *Post*; r = 0.837 *F-up*);
- Cervical and tibial areas: r = 0.795, *p* < 0.001 (r = 0.798 *Pre*; r = 0.710 *Post*; r = 0.831 *F-up*);
- Hand and tibial areas: r = 0.858, *p* < 0.001 (r = 0.766 *Pre*; r = 0.917 *Post*; r = 0.892 *F-up*).

**Figure 4:**
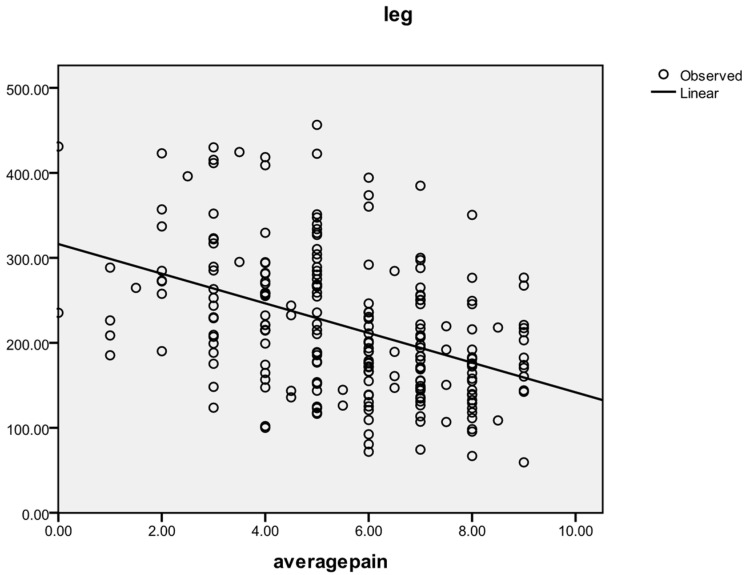
Correlation between average pain level and leg area pain threshold.

A reverse correlation was found between *the average pain level and the pain tolerance measured by algometry* for each of the three areas: *cervical* (r = −0.412, *p* < 0.001; r = −0.222 at *Pre*, r = −0.518 at *Post*, r = −0.421 at *F-up*), *hand* (r = −0.380, *p* < 0.001; r = −0.254 at *Pre*, r = −0.465 at *Post*, r = −0.446 at *F-up*), and *tibial* (r = −0.426, *p* < 0.001; r = −0.320 at *Pre*, r = −0.507 at *Post*, r = −0.470 at *F-up*). The general correlating tendency between the average pain threshold and the leg area pain threshold is shown in Figure 4.

Regarding the impact of pain assessed by the *WHYMPI* test, the most significant differences were observed in Group IV. In terms of *time progression* for these patients, the most notable difference before (*Pre*) and after non-pharmacological therapy (*Post*) was observed for pain severity (*p* = 0.009).

*Inter-group differences*: When comparing patients in Group IV to those in Group II, significantly improved outcomes were observed for the variables of interference and pain severity (*p* < 0.001), along with other significant decreases in life control (*p* = 0.02) and affective distress (*p* = 0.003). Detailed data for all sections of Part I of the inventory at all evaluation moments are presented in Table 6.

## 4. Discussion

It is well-known that FM negatively impacts the patient’s quality of life, resulting in a health status that is poorer than that of individuals with rheumatoid arthritis or osteoarthritis, and comparable to those with severe pulmonary disorders and insulin-dependent diabetes mellitus [37]. Characterized by hyperalgesia, the syndrome is often accompanied by multiple unexplained organic symptoms, as well as anxiety and depression, significantly affecting day-to-day life [37]. Other factors, such as decreased or poor-quality sleep, can also act as triggers for nociception, with an elevation in serum concentrations of interleukin 6 [38]. Chronic stress and psychological factors, especially those accumulated during the COVID-19 pandemic, can lead to additional neurogenic inflammation in FM patients [39]. On the other hand, many patients who have recovered from COVID-19 continue to experience a range of sequelae that impact their daily quality of life [40]. Combining CBT and KT has been shown to be particularly effective in treating several FM symptoms, including pain [41].

In the present study, evaluating pain on a 0–10 numeric scale, the application of non-pharmacological therapy, represented by cognitive therapy and KT, demonstrated the best results six months after treatment completion in patient groups who had not experienced acute COVID-19 infection prior to inclusion in the study, regardless of their vaccination status (*p* < 0.001). The effects of COVID-19 infection on rheumatological patients are still not fully understood [42], and even less is known about the long-term repercussions of what is considered to be a recovered infection in these patients. An Iranian research group [43], studying patients with rheumatological conditions, concluded that these patients have an increased vulnerability to infection due to their underlying disease, which may also aid in managing the consequences of COVID-19. Moreover, symptoms of FM have been reported to worsen during COVID-19 infection [16]. In a recent study (2024), Blanchard et al. demonstrated that persistent FM-like symptoms have been increasingly reported following viral infections, including SARS-CoV-2, with about 30% of the patients with post-COVID-19 syndrome meeting the criteria for FM [44]. Our results support the authors’ conclusion. Comparing the four groups of patients at various moments, while initial pain perception showed no significant differences, by the end of the non-pharmacological therapy, both unvaccinated patients and vaccinated patients who had not experienced acute COVID-19 showed a significant reduction in perceived pain (*p* < 0.01) compared to the two groups of patients who had contracted the illness.

Up to now, little is known about whether autoimmune phenomena occur after the BNT162b2 vaccine. This type of vaccine has been associated with an increased incidence of new cases of myocarditis [45], multiple sclerosis [46], systemic lupus erythematosus [47], connective tissue diseases, and inflammatory arthritis [48]. FM is considered a combination of physical, psychological, and social disabilities, the so-called biopsychosocial model [49]. Regarding FM patients and the perception of pain symptoms, the results of this study support the effectiveness of vaccination. FM patients are known to have certain psychological profile characteristics [50,51]. Vaccination might provide an additional sense of security regarding the disease’s progression and pain perception, even though FM patients reported a higher frequency of adverse events after SARS-CoV-2 vaccination compared to healthy controls [52]. The support for the effectiveness of vaccination as an additional protective factor is also demonstrated by the evolution of maximum pain values perceived by patients and expressed on the VAS scale, both at the end of the treatment application and six months later. These differences were highly statistically significant (*p* < 0.001) in comparison with patients who did not experience acute illness but were not vaccinated.

Regarding algometry and the evolution of pain assessed by this method, the most significant results of the study pertain to the group of vaccinated patients without a history of COVID-19. Although this method is conclusive and commonly used in FM patients [27,28], we did not find any studies in the literature that evaluated pain perception in post-COVID-19 FM patients using algometry. In the present study, concerning the differences among the studied groups, vaccinated non-COVID-19 patients showed a statistically significant increase in pain threshold as measured by algometry (*p* < 0.001). This was true whether they were compared with patients who had experienced acute illness (regardless of vaccination status) or with unvaccinated non-COVID-19 patients. When comparing patients with a history of acute illness, this study did not demonstrate significant differences at any evaluation points (post-treatment or six months later), regardless of vaccination status. Using the subjective evaluation of the effectiveness of cognitive therapy combined with kinetic treatment on pain, as measured by the WHYMPI questionnaire, the same group of patients who had not experienced acute illness and had been vaccinated was the only one to show a statistically significant improvement. Both in the short term, immediately after the end of the non-pharmacological program, and six months after the end of the treatment, the pain was favorably influenced. Both the pain threshold determined by algometry and the results of the questionnaire applied to patients support the findings from before the COVID-19 pandemic [53,54].

Despite these findings, the current study presents some limitations. The lack of research on evaluating FM symptoms after the COVID-19 pandemic posed a limitation for the comparison in the Section 4 and presented a significant challenge. Additionally, including patients from a single university center and vaccination with only one type of vaccine could also represent another limitation of the presented study. A potential additional limitation may be the lack of information regarding specific fibromyalgia treatments, as the study did not account for this aspect. Regarding internal validity of the present study, we consider that taking into account factors such as the baseline medication for FM or other conditions that could influence symptom progression under treatment, which were included as criteria for inclusion or exclusion in this study, lower the internal bias. Additionally, the comparable size of patient cohorts, the inclusion of only adult patients, and evaluations performed by the same specialists throughout the study support the internal validity of the obtained results. Furthermore, we support the external validity based on the premise that a significant portion of the global population has either contracted COVID-19 or has been vaccinated.

## 5. Conclusions

Even if we consider the COVID-19 pandemic to be a thing of the past, patients with FM who experienced SARS-CoV-2 infection may represent a particular group. Thus, while the exact mechanisms are not fully understood, the response to non-pharmacological treatment could be influenced by a history of COVID-19. On the other hand, the study supports the idea that for patients with FM, being vaccinated against COVID-19 should not be expected to impact the therapeutic response to kinesiotherapy or cognitive therapy, as the results of these treatments are comparable to those obtained in patients who have not experienced COVID-19 and have not been vaccinated. Despite the noted limitations, this study suggests that, in addition to these general factors, particular factors must be considered when establishing a treatment plan for patients and predicting pain symptoms. Based on the experience of this study, we can offer some suggestions for future research. However, further robust research is necessary to confirm that experiencing COVID-19 or having a vaccinated status may impact the response to various treatment forms in conditions not as fully understood as FM.

## Figures and Tables

**Table 1:** Baseline characteristics of the studied patients.

Variables	Group I *FM Non-COV*, *Non-Vacc*	Group II *FM COV*, *Vacc*	Group III *FM COV*, *Non-Vacc*	Group IV *FM Non-COV*, *Vacc*	*p*-Value
*N*	30	30	29	31	Ns
Female, *n* (%)	29 (96.6%)	28 (93.3%)	27 (93.1%)	29 (93.5%)	>0.05
Age, years, median (SD, range)	42.4 ± 12.3 (27–53)	43.3 ± 14.5 (28–56)	43.6 ± 11.4 (26–54)	44.3 ± 17.0 (25–54)	>0.05
Primary education, *n* (%)	1 (3.3%)	3 (10.0%)	4 (13.7%)	4 (12.9%)	>0.05
Secondary education, *n* (%)	14 (46.6%)	15 (50.0%)	13 (44.8%)	15 (48.3%)	>0.05
Higher education, *n* (%)	15 (50.0%)	2 (6.6%)	12 (41.3%)	12 (38.7%)	0.03
Currently working, *n* (%)	18 (60.1%)	4 (13.3)%	4 (13.7%)	5 (16.1%)	0.02
Unemployment, *n* (%)	7 (23.3%)	6 (20.0)%	6 (20.6%)	7 (22.5%)	>0.05
Medically retired, *n* (%)	5 (16.6%)	20 (66.6)%	19 (65.5%)	3 (9.6%)	0.02

*n* = number of patients; non-COV = without COVID-19 infection; non-Vacc = unvaccinated patients; Vacc = vaccinated patients; COV = COVID-19 infection.

**Table 2:** Clinical diagnosis data.

Clinical Data	Group I *FM Non-COV*, *Non-Vacc* *n* = 30	Group II *FM COV*, *Vacc* *n* = 30	Group III *FM COV*, *Non-Vacc* *n* = 29	Group IV *FM Non-COV*, *Vacc* *n* = 31	*p*-Value
Years since FM diagnosis, mean ± SD	7.3 ± 3.9	6.9 ± 4.1	7.1 ± 3.5	6.9 ± 4.6	>0.05
Duration of pain before FM diagnosis, years mean ± SD	6.1 ± 9.7	5.8 ± 7.3	6.3 ± 8.1	5.7 ± 11.6	>0.05
Irritable bowel syndrome, %	21.8%	23.5%	20.0%	28.1%	>0.05
Chronic fatigue syndrome, %	18.7%	20.6%	33.4%	22.5%	>0.05
Tension headaches, %	53.1%	52.9%	56.6%	53.1%	>0.05
Endocrine disorders, %	43.7%	41.2%	40.0%	40.6%	>0.05
Rheumatic disorders, %	21.8%	23.5%	23.3%	25.0%	>0.05
Anxiety or depression, %	59.3%	52.9%	50.8%	41.2%	>0.05
Entry 2 sleep disturbances, %	46.8%	44.1%	43.3%	41.8%	>0.05

*n* = number of patients; SD = standard deviation; non-COV = without COVID-19 infection; non-Vacc = unvaccinated patients; Vacc = vaccinated patients; COV = COVID-19 infection.

**Table 3:** Average pain rated on a numeric scale (VAS).

Average Pain	*Pre*, Mean (SD)	*Post*, Mean (SD)	*F-up*, Mean (SD)	*Pre/Post* (*p*-Value)	*F-up/Post* (*p*-Value)	*F-up/Pre* (*p*-Value)
Group I *FM non-COV, non-Vacc,* *n* = 30	7.2 ± 1.2	5.6 ± 1.5	6.2 ± 1.7	0.000	0.000	0.000
Group II *FM COV, Vacc,* *n* = 30	7.1 ± 1.2	6.7 ± 1.4	7.0 ± 1.5	0.4	0.7	0.7
Group III *FM COV, non-Vacc*, *n* = 29	6.5 ± 1.7	5.8 ± 1.7	6.2 ± 1.7	0.08	0.6	0.4
Group IV *FM non-COV, Vacc*, *n* = 31	6.0 ± 1.4	3.4 ± 1.6	4.0 ± 1.7	0.000	0.1	0.000

*n* = number of patients; SD = standard deviation; non-COV = without COVID-19 infection; non-Vacc = unvaccinated patients; Vacc = vaccinated patients; COV = COVID-19 infection.

**Table 4:** Pain tolerance in all measured areas of the body.

Group	Moment	CervicalR	CervicalL	HandR	HandL	TibialR	TibialL
Group I *FM COV*, non-Vacc Mean (SD)	*Pre*	90.0 ± 35.3	85.8 ± 32	143.0 ± 67.0	109.8 ± 46.5	189.5 ± 98.9	174.5 ± 77
	*Post*	81.3 ± 42.7	84.6 ± 40.3	125.7 ± 58.9	112.3 ± 64.7	151.4 ± 65.8	149 ± 59.6
	*F-up*	76.7 ± 28.5	84.2 ± 41.8	123.2 ± 48.3	128 ± 65.5	161 ± 82.5	157.7 ± 62.5
Group II *FM COV*, Vacc Mean (SD)	*Pre*	94.1 ± 40.4	102.1 ± 52.6	155.7 ± 50.7	134.4 ± 52.9	213.1 ± 86.5	204.6 ± 75
	*Post*	67.3 ± 38.1	76.1 ± 45.9	103.9 ± 59.1	101.5 ± 54.6	163.3 ± 87.2	153.4 ± 79
	*F-up*	103.9 ± 49.2	105.6 ± 49.0	141.0 ± 56.6	127.2 ± 64.5	189.1 ± 83.7	182.6 ± 68.8
Group III *FM COV*, non-Vacc Mean (SD)	*Pre*	111.3 ± 24.7	104.0 ± 25	144.5 ± 44.4	145 ± 39.7	174.5 ± 36.1	164.0 ± 33.6
	*Post*	114.4 ± 28.7	110.0 ± 28.1	139.6 ± 40.5	141.2 ± 39.1	204.8 ± 92	155.6 ± 42.2
	*F-up*	114.1 ± 28.2	108.3 ± 28.7	141.5 ± 41.6	139.4 ± 38.1	173.3 ± 34.5	157.9 ± 34.2
Group IV FM non-COV, Vacc Mean (SD)	*Pre*	152.0 ± 38.7	156.0 ± 35.0	207.5 ± 48.0	199.4 ± 43.1	263.2 ± 59.0	260.5 ± 70.7
	*Post*	169.8 ± 45.4	171.7 ± 40.0	221.7 ± 43.2	217.9 ± 54.3	292.9 ± 79.3	288.8 ± 75.6
	*F-up*	162.3 ± 35.5	156.1 ± 28.5	219.9 ± 41.3	192.5 ± 33.8	307.8 ± 63.3	305.3 ± 66.4

SD = standard deviation; R = right; L = left; non-COV = without COVID-19 infection; non-Vacc = unvaccinated patients; Vacc = vaccinated patients; COV = COVID-19 infection.

**Table 5:** The *p*-values in Post and Follow-up moments between studied patient groups.

Moment	Group	Cervical (R) (p)	Cervical (L) (p)	Hand (R) (p)	Hand (L) (p)	Tibial (R) (p)	Tibial (L) (p)
*Post*	Group I/II	0.000	0.001	0.009	0.002	0.078	0.895
*Post*	Group I/III	0.001	0.007	0.297	0.353	0.013	0.626
*Post*	Group I/IV	0.000	0.000	0.000	0.000	0.000	0.000
*Post*	Group II/III	0.181	0.446	0.154	0.090	0.551	0.807
*Post*	Group II/IV	0.000	0.000	0.000	0.000	0.000	0.000
*Post*	Group III/IV	0.000	0.000	0.000	0.000	0.000	0.000
*F-up*	Group I/II	0.333	0.797	0.969	0.380	0.349	0.087
*F-up*	Group I/III	0.000	0.013	0.125	0.056	0.461	0.988
*F-up*	Group I/IV	0.000	0.000	0.000	0.000	0.000	0.000
*F-up*	Group II/III	0.011	0.154	0.192	0.371	0.192	0.145
*F-up*	Group II/IV	0.000	0.000	0.000	0.000	0.000	0.000
*F-up*	Group III/IV	0.000	0.000	0.000	0.000	0.000	0.000

Group I: *FM non-COV*, *non-Vacc*; Group II: *FM COV*, *Vacc*; Group III: *FM COV*, *non-Vacc*; Group IV: *FM non-COV*, *Vacc*; R-right; L-left.

**Table 6:** WHYMPI scores.

Group IV vs. Group II	*Pre* Mean (*p*-Value)	*Post* Mean (*p*-Value)	*F-up* Mean (*p*-Value)	*Pre/Post* (*p*-Value)
Interference	3.40 (0.03)	2.90 (0.000)	3.20 (0.006)	0.04
Support	3.70 (0.3)	3.62 (1)	3.72 (0.6)	1
Pain severity	3.84 (0.3)	3.07 (0.000)	3.66 (0.05)	0.009
Life control	3.50 (0.2)	4.10 (0.02)	3.90 (0.4)	0.2
Affective distress	3.10 (0.1)	2.62 (0.003)	2.83 (0.09)	0.5

WHYMPI = West Haven-Yale Multidimensional Pain Inventory.

## Data Availability

Data are contained within the article.
